# Introducing the Team Card: Enhancing governance for medical Artificial Intelligence (AI) systems in the age of complexity

**DOI:** 10.1371/journal.pdig.0000495

**Published:** 2025-03-04

**Authors:** Lesedi Mamodise Modise, Mahsa Alborzi Avanaki, Saleem Ameen, Leo A. Celi, Victor Xin Yuan Chen, Ashley Cordes, Matthew Elmore, Amelia Fiske, Jack Gallifant, Megan Hayes, Alvin Marcelo, Joao Matos, Luis Nakayama, Ezinwanne Ozoani, Benjamin C. Silverman, Donnella S. Comeau

**Affiliations:** 1 Center for Bioethics, Harvard Medical School, Boston, Massachusetts, United States of America; 2 Department of Radiology, Beth Israel Deaconess Medical Center, Boston, Massachusetts, United States of America; 3 Department of Biomedical Informatics, Harvard Medical School, Harvard University, Boston, Massachusetts, United States of America; 4 Tasmanian School of Medicine, College of Health and Medicine, University of Tasmania, Hobart, Tasmania, Australia; 5 Laboratory for Computational Physiology, Massachusetts Institute of Technology, Cambridge, Massachusetts, United States of America; 6 Division of Pulmonary, Critical Care, and Sleep Medicine, Beth Israel Deaconess Medical Center, Boston, Massachusetts, United States of America; 7 Department of Biostatistics, Harvard T.H. Chan School of Public Health, Boston, Massachusetts, United States of America; 8 Faculty of Medicine, The Chinese University of Hong Kong, New Territories, Hong Kong SAR; 9 Indigenous Media in Environmental Studies Program and the Department of Data Science, University of Oregon, Eugene, Oregon, United States of America; 10 Duke Health, AI Evaluation and Governance, Duke University, Durham, North Carolina, United States of America; 11 Department of Preclinical Medicine, Institute of History and Ethics in Medicine, TUM School of Medicine and Health, Technical University of Munich, Bavaria, Germany; 12 Department of Critical Care, Guy’s and St. Thomas’ NHS Trust, London, United Kingdom; 13 Department of Environmental Studies, University of Oregon, Eugene, Oregon, United States of America; 14 Medical Informatics Unit, College of Medicine, University of the Philippines Manila, Philippines; 15 Faculty of Engineering, University of Porto, Portugal; 16 Institute for Systems and Computer Engineering, Technology and Science, Porto, Portugal; 17 Department of Ophthalmology, Sao Paulo Federal University, Sao Paulo, Brazil; 18 Machine Learning and Ethics Research Engineer, Innovation n Ethics, Dublin, Ireland; 19 Department of Human Research Affairs, Mass General Brigham, Somerville, Massachusetts, United States of America; 20 Institute for Technology in Psychiatry, McLean Hospital, Belmont, Massachusetts, United States of America; National Tsing-Hua University: National Tsing Hua University, TAIWAN

## Abstract

This paper introduces the Team Card (TC) as a protocol to address harmful biases in the development of clinical artificial intelligence (AI) systems by emphasizing the often-overlooked role of researchers’ positionality. While harmful bias in medical AI, particularly in Clinical Decision Support (CDS) tools, is frequently attributed to issues of data quality, this limited framing neglects how researchers’ worldviews—shaped by their training, backgrounds, and experiences—can influence AI design and deployment. These unexamined subjectivities can create epistemic limitations, amplifying biases and increasing the risk of inequitable applications in clinical settings. The TC emphasizes reflexivity—critical self-reflection—as an ethical strategy to identify and address biases stemming from the subjectivity of research teams. By systematically documenting team composition, positionality, and the steps taken to monitor and address unconscious bias, TCs establish a framework for assessing how diversity within teams impacts AI development. Studies across business, science, and organizational contexts demonstrate that diversity improves outcomes, including innovation, decision-making quality, and overall performance. However, epistemic diversity—diverse ways of thinking and problem-solving—must be actively cultivated through intentional, collaborative processes to mitigate bias effectively. By embedding epistemic diversity into research practices, TCs may enhance model performance, improve fairness and offer an empirical basis for evaluating how diversity influences bias mitigation efforts over time. This represents a critical step toward developing inclusive, ethical, and effective AI systems in clinical care. A publicly available prototype presenting our TC is accessible at https://www.teamcard.io/team/demo.

## Author summary

Bias in medical artificial intelligence (AI) systems refers to systematic errors that unfairly favor or disadvantage certain groups or outcomes. While often attributed to data quality issues, such as incomplete or unrepresentative datasets, bias also stems from the unexamined assumptions of researchers and developers. These influences, known as positionality, include the unique backgrounds, experiences, and worldviews that shape decisions during AI development. When unaddressed, positionality can lead to inequitable outcomes and suboptimal performance in healthcare. The Team Card (TC) is a tool designed to address these challenges by providing a structured framework for reflecting on and documenting positionality variables, perspectives, and biases throughout AI development. By integrating diverse perspectives, the TC helps research teams identify and address blind spots in development practices that stem from researchers’ subjectivity. It also enables teams to assess whether addressing positionality improves fairness and reduces bias in the performance of AI in clinical settings. Research across fields such as business, science, and organizational behavior highlights that diversity fosters better decision-making, enhanced innovation, and improved overall outcomes. A publicly available TC prototype, reflecting our Team Card, is accessible at https://www.teamcard.io/team/demo, offering a practical, adaptable solution for advancing equitable and effective AI systems in healthcare.

## 1. Introduction

Over 40% of hospitals in the United States have integrated Clinical Decision Support (CDS) software into a broad spectrum of critical functions including diagnostics, disease management, prescription services, and alarm systems, significantly enhancing healthcare delivery and patient care [1]. Despite the challenges presented by factors such as compatibility with existing clinical infrastructure [2], data bias [3] and ethical concerns [4,5,6], CDS software functions employing machine learning (ML) algorithms and other forms of artificial intelligence (AI) currently represent a rapidly expanding use case for AI in healthcare. These AI-driven CDS software functions (medical AI systems) are often described as non-knowledge based, because they generate recommendations through statistical analysis or pattern recognition within electronic health records and other data sources, instead of relying on established medical knowledge [1]. This raises concern for the explainability of clinical recommendations provided by medical AI systems, which can sometimes function as “black-boxes”, meaning that the resulting decisions are not easily understood [4,7]. The increasing deployment of medical AI systems has thus prompted action to establish a basis of trust in these tools by improving their accuracy, robustness, and explainability [8]. To address these concerns in the United States, the Biden administration issued an executive order in October of 2023 on the trustworthy development and safe use of AI [9]. In healthcare, the order calls for the development of an AI assurance policy that encompasses a safety program and relevant standards. This follows earlier action by both the Food and Drug Administration (FDA) and the Department of Health and Human Services (DHHS), in response to calls to regulate the growing prevalence of algorithmic decision making in patient care.

In 2019 [10] and 2022, [11] the FDA issued draft and final guidance, respectively classifying, certain CDS software, clarifying which functions of CDS qualify as Software as a Medical Device (SaMD) subject to its regulatory oversight. According to the guidance, high-risk CDS software functions that are intended to inform clinical decisions and that meet the FDA’s definition of a medical device must undergo rigorous premarket review, including the evaluation of supporting clinical data to ensure safety and effectiveness. However, many medical AI systems do not meet the criteria for SaMD and therefore fall outside the FDA’s regulatory jurisdiction [12,13]. Furthermore, among the medical AI systems that do qualify as devices, the majority are deemed to present either low risk or substantial equivalence to previously authorized devices, allowing them to gain clearance through expedited pathways, such as the 510(k) process, which often exempts them from requiring clinical review [14,15].

In 2022, the DHHS proposed to update existing provisions of the Affordable Care Act that prohibit discrimination in covered health programs to also prohibit discrimination by clinical algorithms [16]. However, some argue that the proposed framework places excessive demands on healthcare professionals, as it requires them to effectively evaluate all algorithms implemented in their practices for potential discrimination across a broad range of parameters including; race, sex, color, national origin, age, and ability. It is also argued that the framework fails to account for the technical expertise required to audit ML algorithms for potential bias [12,13]. Finally, there is also concern that the proposed standard may simply be unachievable; not only are the algorithms powering medical AI systems typically proprietary and thus inaccessible [17], but also, these algorithms and their results may not be explainable [4,7,18]. In response to these challenges, the American Medical Association has stressed the importance of establishing crosscutting responsibilities for developers and end users of medical AI systems [19], highlighting the need for greater adherence to consensus-driven AI assurance standards [20] further upstream - among those who develop medical AI systems.

Current upstream efforts to avert harmful bias in medical AI systems have focused on better contextualization of the performance characteristics of ML algorithms through data and code sharing [21]. However, a researcher’s positionality coincides with biases at every stage of development – not only in the acquisition of data and the development of ML algorithms, but also in the assessment of the resulting medical AI system’s performance [22,23]. It thus becomes imperative to situate the positionality of individuals developing these systems, by accounting for potential biases that may stem from researcher subjectivity, throughout the AI development lifecycle. To date, the most significant efforts to situate ML researchers have been limited to scientometrics, which report on characteristics such as the regional location or origin of authors who have works published in scientific journals. While this approach provides some insight [24], there remains no standardized method for communicating the situatedness, as well as the potential resulting biases, of the individuals behind the development of a given medical Al system.

This work introduces the Team Card (TC); an ethical protocol that narrates researcher identity and positionality as essential components of AI development. The protocol assists research and development teams to critically examine their positionality in relation to the AI they create, thereby mitigating the encoding of harmful bias in medical AI systems. The TC fosters epistemic diversity and inclusion by encouraging the incorporation of orthogonal perspectives in development practices while also promoting accountability by directly linking medical AI systems to their creators. Additionally, the TC provides a structured framework for collecting the data needed to validate diversity’s impact on the mitigation of bias in the research and development of medical AI systems.

## 2. Positionality and epistemic diversity in the research and development of AI

Discussions of positionality in research derive from the premise that scientific knowledge is neither value-neutral nor objective. Rather, it is socially situated and is laden with both values and intent, which serve to reinforce the dominance of established views [25] and hierarchies of power [26]. *Dominant Science*, as observed by feminist and Indigenous scholars, assesses knowledge against its own self-image. In accepting – as scientific – those knowledges that align with established forms of power and eschewing those that do not, it engenders oppressive characteristics [27,28] that presume to adjudicate who can engage in the production of scientific knowledge [29].

Positionality broadly refers to an individual’s worldview, which is shaped by gender, ethnic identity, experiences, social milieu, cultural background, and other formative influences. The term has become increasingly central to qualitative research processes, serving to identify a method for quality control [30] by locating the researcher in the scientific process. Positionality statements in research are a mode of reflection on how the researcher’s perspective might have shaped the design, implementation, and analysis of the project, illuminating how the situated subjectivity of knowledge can influence outcomes at every stage of the research process. Positionality statements thus communicate the researcher’s orientation toward the project through the lens of that individual’s worldview and within the context of a broader social milieu.

In this manner, positionality statements engage an ethic of critical reflexivity as a necessary precursor to addressing harmful social biases in scientific knowledge [25]. Current efforts to situate knowledge in research are informed by established standards for qualitative reporting, such as Standards for Reporting Qualitative Research (SRQR) and Consolidated Criteria for Reporting Qualitative Research (COREQ), which aim to transparently convey reflexivity and details about research teams vis a vis qualitative studies. For example, Indigenous scholars have underscored the importance of locating themselves through explicit self and cultural identification with protocols of introduction [31]. Similarly, others have described how by sharing their backgrounds and life experiences, they seek to build trust between researchers and the researched [32], in a process of relational accountability [33].

Developing these ideas, feminist and care-oriented approaches have proposed ethical frameworks for the research and development of AI that begin from the proposition that AI is socially situated and that the data with which ML algorithms are trained are not neutral [34]. These frameworks support research methods [35] that focus on diversity and empowerment to mitigate the potential for representational harm in the AI development lifecycle. They do so by contending that a diversity of perspectives should shape the design and development of the technologies that define society, ensuring that these technologies are attuned to intersecting oppressions such as racism [36], sexism, and classism [34].

Nevertheless, the AI research and development landscape continues to lack diversity [37]. A scientometric analysis of original research in medical AI system development indicates that a concentration of relevant academic papers emanates from distinct knowledge hubs in the US and in China [38]. This centrality of authorship establishes collaboration and co-authorship dynamics that serve to benefit scientific productivity, but also result in increasingly homogenous research [39] that reflects the positionality of certain research groups to the exclusion of others. The resulting research is vulnerable to potential blind spots; its efficiency and proliferation derives from homogenous research teams that tend toward convergent team processes, quickly aligning on objectives and conclusions [40].

By contrast, divergent team processes that juxtapose differing values and ideas are a hallmark of ethnically diverse teams [41]; studies show that researchers think differently in ethnically diverse groups because they anticipate divergent team process to more rigorously challenge their ideas and to alter small group dynamics [42,43]. The observed result is that socially diverse teams – characterized by greater diversity in ethnicity, gender, age, and institutional affiliation – tend to produce research that is more frequently cited, with socio-ethnic diversity in authoring teams having the greatest impact on increased citations [42,44]. Cognitive science and organizational behavior also demonstrates that diverse teams – that include different kinds of thinkers from varying disciplines – outperform homogenous groups on complex tasks and are better equipped to identify blind-spots, because they bring varied perspectives and approaches to problem solving [44,45,46,47].

Beyond research, diversity delivers tangible benefit across multiple domains. In clinical practice, a more diverse workforce has been shown to improve diagnostic accuracy and outcomes across patient populations [48]. In business, research shows that organizations with more diverse management teams demonstrate higher levels of innovation, better decision-making and achieve superior operating performance due to the variety of perspectives and experiences they bring to problem-solving [49,50,51,52]. Similarly, in governance, nations with inclusive political and economic institutions are seen to be more prosperous [53]. These findings highlight the value of diversity in fostering productive, adaptive, and equitable systems.

Drawing these perspectives together through practical experimentation, we observe that while the social diversity of the authoring team brought a range of situated perspectives to this research, it did not inherently lead to epistemic diversity – the diversity of ways of thinking, knowing, and problem-solving. This distinction is critical for advancing bias mitigation in AI. In our experience, epistemic diversity must be cultivated through an active and collaborative process of critical self-reflection, interdisciplinary engagement, and the contrasting of positionalities within research teams. The TC protocol enables teams to systematically characterize positionality variables by creating structured spaces for reflection on how positionality shapes methodological choices in AI development. This framework allows teams to identify factors that may influence bias, such as social identities, disciplinary backgrounds, and lived experiences. By enabling the documentation and analysis of these variables, the TC helps uncover patterns that contribute to bias, offering actionable insights for its mitigation. This process is particularly valuable for addressing biases in medical AI, which are intersectional in nature and arise from a broad range of factors spanning data quality and model design, as well as social and technological determinants [54,55,56].

While it is reasonable to assume that non-diverse teams may overlook biases due to homogeneity in perspectives, our argument is supported by evidence that diverse teams, particularly those fostering epistemic diversity, are better positioned to identify blind spots – which is essential for addressing the complex nature of bias in AI.

## 3. The importance of representation in the research and development of medical AI systems

The capacity for AI to perpetuate prejudice in healthcare is increasingly recognized. Bias in clinical algorithms and medical AI systems has engendered discrimination across a range of demographic variables including race and ethnicity, age, disability, socio-economic status, English language proficiency and gender [57,58,59]. Attention to health disparities in the US has largely focused on racial and ethnic disparities in patient outcomes [60]. These disparities have rooted across all stages of the clinical value chain, and are seen to affect the lives of millions of patients across the US [61]. The issue is most acute in medical diagnostics where, despite a contemporary understanding that race is not a dependable proxy for genetic difference, old beliefs to the contrary remain embedded in medicine. This is evident in the longstanding practice of “correcting” diagnostic algorithms for race. Ubiquitous diagnostic algorithms and clinical practice guidelines that adjust outputs for race include examples in cardiology, nephrology, obstetrics, oncology, endocrinology, pulmonology, and urology [62]. Explicit racial biases in healthcare – such as the aforementioned race corrections – mingle with implicit racial biases arising from learned attitudes, to produce the increasing number of AI failures observed along color lines in clinical settings. The expanding corpus of racial discrimination in medical AI systems spans diagnostic procedures, therapeutic interventions and facility management systems [61,62,63,64,65].

It should be noted that these disparate patient outcomes are typically attributed to deficiencies in the data used to train medical AI systems. The issue of data quality includes situations in which the data do not reflect the true epidemiology of a demographic due, for instance, to historic racial bias in diagnosis [4,66,67]. It also includes situations where unequal access to care has been encoded as statistical forms of bias, as well as situations with sampling concerns - where data sets do not contain enough demographic diversity. These limitations decrease the reliability of resulting medical AI systems in racially diverse settings and are, to varying degrees, the result of existing social bias and inequality. The true scope of the issue however extends beyond data quality, as AI development practices are themselves susceptible to the embedded beliefs and unconscious judgments of research and development teams.

Isolating the locus of harmful bias in medical AI systems is made more complex where there are several digital determinants of health that are socially situated and that will affect system performance. Practical examples of these determinants in the clinical setting might include language concordance, digital literacy, and access to digital infrastructure such as electricity and stable internet connectivity [56]. It can thus be understood that harmful bias in medical AI systems is integrally related to forms of social bias: discriminatory patient outcomes result when existing forms of social inequality are normalized by researchers and encoded into data. These biased outcomes further disadvantage communities that have already been structurally marginalized, so that forms of social bias and statistical bias in AI interact with each other in a recursive manner.

Emerging research on the autodidactic nature of certain medical AI systems presents further considerations: Wawira Gichoya et al. demonstrated that when ML is applied to de-identified medical images such as radiographs, CT scans, and mammograms, it can predict a subject’s self-identified racial identity with an accuracy of 80-99% across these imaging modalities [68]. The capability is readily acquired by standard ML algorithms that are trained with diverse data sets and accuracy persists, even after controlling for historic racial proxies like body-mass index, disease distribution and breast density.

These medical AI systems can accurately predict a patient’s race from medical images that are corrupted, noisy, or cropped. Moreover, they base racial inferences on data that lies beyond the standard medical variables utilized by radiologists and other health care professionals. Thus, physicians may be unable to monitor and control this behavior when it is undesirable. Additionally, since many models are built using de-identified data sets, the inherent bias of these models may not be readily identifiable. Set against a deep legacy of racism in the U.S. healthcare system [69], the latent ability of medical AI systems to make unprompted racial inferences is of serious concern. The same phenomenon has also been observed with gender inferences, where an AI system reading only de-identified retinal images was found to accurately predict multiple cardiovascular risk factors, including gender, even though ophthalmologists could not [6,70].

These findings highlight the immense scope for medical AI systems to deepen disparate patient outcomes along social divides, particularly where models can make undetected inferences from a vast mosaic of data. It is thus crucial to facilitate the development of equity-focused medical AI systems by both empowering research and development teams to identify and address the harmful effects of social bias in AI development practices and by holding teams accountable for the differential impact of their AI products [71], thereby ensuring that vulnerable patient populations are not further marginalized by rapid improvements in health innovation [72].

## 4. Geopolitical considerations for equity in medical AI system development

A primary issue facing the upstream segment of the AI ecosystem is the matter of data representativeness, as the preponderance of training data currently originates from the United States and China. A 2022 analysis of PubMed publications reveals that c.40% of the datasets referenced in medical AI literature are sourced from the United States and c.14% from China [73]. This leads to the development of solutions that generalize less effectively to underrepresented groups. A further concern is the issue of dominant groups in the research and development of AI as interwoven social and professional networks are prevalent in the medical research community, establishing and entrenching niche dominance [74]. There is a distinct lack of diversity within these niches as researchers with higher importance and centrality within their respective niches are seen to be less likely to be female or to come from low-and-middle-income countries (LMICs) [55,75,76].

The resulting centralization of knowledge production in high-income countries has created a power imbalance, where these regions have disproportionate influence on global standards and policies related to AI products. Moreover, the emerging disconnect between AI developers and the contexts in which their products are deployed means that there may be no process of accountability by which to address situations in which AI products cause harm – particularly when products from high-income countries are exported to other regions. International collaboration among regulatory bodies to intensify scrutiny on the datasets used to train medical AI systems, particularly when these systems are deployed in diverse geographic and social contexts [71], is one part of the solution. A comprehensive solution, however, also requires a focus on contextual testing to ensure that AI products are appropriate for the contexts in which they are deployed. This goal can be achieved by leveraging a growing global network of AI research groups through international partnerships. International collaboration among research groups not only fosters diversity and inclusion in medical AI system development by encouraging data exchange and open science practices, but also presents opportunities for capacity building in LMICs.

Although the AI landscape currently lacks coordinated regulation [77], the ecosystem has sought to adopt various initiatives that support a standard of trustworthiness for AI. Successive waves of self-regulation have seen the adoption of Datasheets for Datasets [78], which improves communication between dataset creators and users. The subsequent adoption of Model Cards [21] was a further step to standardize the disclosure of key performance characteristics for trained ML algorithms. These cards detail operating parameters, such as intended use cases and performance evaluation criteria, which helps to reduce the deployment of trained ML algorithms in scenarios for which they are not adequately designed. Algorithm assurance [79] through model audits has also been proposed as an IT risk management protocol, and the growing number of audits signals a commitment to the development of equity focused AI [22]. However, more is required to realize a standard of trustworthiness for AI in healthcare.

## 5. Core attributes of the TC

TCs can provide relevant information about the authorship of medical AI systems. While they are not intended to be prescriptive, they should include core information that enables stakeholders to understand salient positionality attributes of the contributors behind a given medical AI system. We note that guidelines established by the SRQR and COREQ have been criticized for being overly rigid and for adopting a focus on checklist detail, rather than retaining focus on the holistic outcomes of relevant disclosures [80]. This protocol considers the shortcomings of comparable frameworks and instead invites teams to nominate the most relevant elements for disclosure in each situation. Written statements, diagrams, illustrations, audio-visual and multi-modal content are all viable media for the expression of positionality in this disclosure protocol.

Core attributes of the TC broadly comprise a discussion of team positionality and disclosure of team composition and are detailed below (Table 1). They can be thought of as a synthesis of two primary tenants of AI assurance and governance: (i) a self-reflective component to support the effective mitigation of harmful bias and (ii) a disclosure component to encourage greater accountability. We maintain that teams, and individuals within them, should retain control over what they elect to disclose and should not be compelled to reveal private aspects of their positionality. We however emphasize that while teams are free to add or omit information as they deem relevant, a culture of transparency is essential for the protocol’s practical success and for fostering more responsible practices in medical AI system development.

**Table 1:** Core attributes of the TC.

CORE ATTRIBUTE		DISCLOSURE PARAMETER	GOVERNANCE STANDARD	GOVERNANCE OBJECTIVES & ACTION STEPS
**Discussion of positionality**		[1] Positionality	Transparency, accountability	A discussion of the perspective from which the problem is being approached, including reflections on appointed methodology and data selection, as well as team composition. Areas of concern for potential harmful bias and the measures taken to mitigate these risks are highlighted
**Disclosure of team composition**	**Team structure**	[2] Roles & expertise	Ethical oversight	Include team functions that retain a focus on ethical considerations
			End-user advocacy	Include perspectives that advocate for the end-user (e.g. physicians and patient advocates)
			Regulatory compliance	Maintain compliance with regulatory standards by including dedicated or consulted compliance functions
		[3] Institutional affiliation	Multi-disciplinary collaboration	Consider cross-disciplinary collaborations to include varied perspectives and expertise
			Regulatory body engagement	Maintain a dialogue with relevant regulatory bodies to enhance compliance functions
			Ethical review	Design AI system development process that anticipates the formal review and approval of an appropriately constituted committee
		[4] Context/ location	Contextual testing and validation	Test and validate AI tools in the geographic contexts in which they will be deployed
	**Team identity**	[5] Identity	Inclusion	Inclusion is dynamic in that it recognizes dimensions of diversity that emerge from identities, as they pertain to team objectives, including the intersection of socioeconomic status, ethnicity, cultural background, gender, ability, and sexual orientation - but goes beyond to ensure belonging, respect, and success. Appropriate governance standards should comprise elements of bias mitigation as well as those that take into account inclusivity in medical AI design Consider modes of team engagement and TC presentation that promote accessibility of the content and accommodate effective collaboration across a potential range of abilities. This might include consideration of formats such as audio and illustration Consider documenting the rationale behind the problem definition and the selection of proxy variables, reflect on how the framing aligns with intended outcomes, evaluate how closely the proxies approximate the true variables of interest, and implement measures to mitigate foreseeable risks

Like the teams they describe, TCs are not intended to remain static. They should be updated periodically to ensure they accurately reflect the evolving composition and positionality of teams. We also recommend that prior contributors, who may no longer be active team members, be appropriately acknowledged to support the fair recognition of contributions over time and to further cultivate a culture of inclusion. Finally, while we believe that TC disclosures should primarily include information typically disclosed in research endeavors, we acknowledge that publicly sharing sensitive personal information may pose privacy risks. Teams should carefully consider these factors when compiling TC disclosures.

## 6. A presentation of our TC

The authoring team experimented with the TC protocol to investigate whether social diversity within our research team could effectively contribute to epistemic diversity and ultimately assist us to interrogate areas of potential bias in the development of this manuscript. Although we did not engage in AI production, our objective was to apply the TC framework experimentally, as a lens for identifying and addressing potential biases in our research. We present the TC compiled by the authors of this manuscript as a tangible example of how the protocol can be interpreted to convey salient information about team composition and the positionality attributes conferred by that composition.

We find that the social diversity of our team, combined with the various disciplines and competences among authors, as well as a shared commitment to reflexive dialogue on how these lenses shape our understanding of bias in AI, together enriched epistemic diversity in our research and manuscript preparation. We posit that this enhanced epistemic diversity could play a meaningful role in supporting effective bias mitigation strategies in the development of medical AI systems, and advocate for the adoption of TCs in other teams aiming to address similar challenges. This approach not only supports bias mitigation but also establishes a foundation for empirically assessing the value of diversity in shaping ethical and effective medical AI systems. Finally, we emphasize that a cultural shift in AI development that shows commitment to meaningful engagement across perspectives, is essential to the scuccess of the TC and similar bias mitigation strategies.

We re-iterate that the TC presented here serves as an example relevant to our specific context—preparing a manuscript on the TC protocol itself—and thus does not include the technical considerations that would apply to AI development. We acknowledge that different interpretations, whether through text, visual, or mixed media, could also effectively implement the protocol. We later discuss two illustrative cases to further demonstrate the protocol’s practical implementation and benefits.

### [1] Discussion of the authoring team’s positionality

This area of research was prompted by the observations of physician-scientists on the team, who noted structural inequality in healthcare outcomes across patient populations. These observations raised concern for the potential deepening of disparities along social fault lines, with the layering of AI on legacy healthcare systems. The diverse positionalities within the team include perspectives informed by lived experiences of intersecting oppressions, providing a nuanced understanding of how individuals may encounter discrimination in healthcare. These perspectives also highlight the influence of social determinants on health outcomes across patient groups. The following discussion synthesizes the positionality statements prepared by individual team members. The statements can be accessed at the following link: https://www.teamcard.io/team/demo.

The authoring team’s positionality reflects the diverse experiences and commitments of a research team dedicated to addressing bias and promoting equity in medical AI systems. Members bring unique, firsthand perspectives from varied global contexts, including clinical experience in the U.S., Brazil, the Philippines, Iran, China, the UK, and Tasmania - which drives the team’s focus on health equity and access for marginalized populations. Others on the team emphasize reflexivity and transparency in AI research, advocating for inclusive methodologies informed by their backgrounds in machine learning, anthropology, and public health. The team’s commitment to ethically responsible AI development is further strengthened by the contributions of members with expertise in bioethics, and feminist philosophy, who focus on mitigating harm and bias in healthcare applications. Additionally, members of the team share a dedication to matters of social justice, with personal and professional experiences of discrimination shaping their advocacy for accountability and governance in the development of medical AI systems. By integrating technical, social, and ethical dimensions, we believe the mix of competencies and perspectives on the team facilitated robust and critical assessment of potential blind spots in the research.

### [2] Roles and expertise

Authorship was engaged collaboratively by a team of 16 individuals, 12 of whom identify as clinicians, ML engineers, data scientists or an intersection of these descriptors. This allowed for self-reporting and advocacy from perspectives across the medical AI value chain, ranging from AI product developers to healthcare professionals implementing medical AI systems as end-users. Remaining authors contributed expertise as anthropologists, social science researchers, bioethicists and healthcare technology investors, each providing an unique lens on the social determinants of equitable AI. Additionally, 2 members of the team contributed ethical review and regulatory compliance considerations, informed by their engagements with institutional review boards.

### [3] Institutional affiliations

Institutional affiliations of the authoring team include Duke University, Harvard Medical School, Mass General Brigham (MGB), Massachusetts Institute of Technology, University of Oregon, Technical University of Munich School of Medicine and Health, the Beth Israel Deaconess Medical Center (BIDMC), and the University of the Philippines Manila. Engagement across the team delivered multi-disciplinary collaboration by integrating expertise across clinical practice, computer science and engineering and the social sciences. Although the preparation of this manuscript is not subject to regulation, direct engagement with research oversight and ethical review bodies was solicited through the contributions of sitting members of the MGB IRB, who form part of the authoring team. We acknowledge that our understanding of the regulatory landscape for medical AI systems could be further strengthened by consulting relevant individuals or bodies as these frameworks evolve. In the interim, we have relied on the appropriate experience of members on the team and desktop research. No funding affiliation is relevant to this manuscript.

### [4] Context/ geographic location

The authoring team’s members are active researchers in Australia, Brazil, Germany, Indonesia, Ireland, Spain, the U.S. and the U.K.. They have each contributed perspectives on harmful bias mitigation that are relevant to the AI research and development ecosystems prevailing in their respective regions, as well as the clinical contexts in which medical AI systems are adopted in these regions. Additionally, authors based outside of their home countries have contributed relevant impressions that attune to the contexts of their countries of origin, which include China, Iran, Nigeria and South Africa. We believe that this geographic diversity enables a rich contextual evaluation of the TC protocol, which we observe to be robust across various institutional settings, spanning academia and industry, in the regions considered.

**Fig 1:**
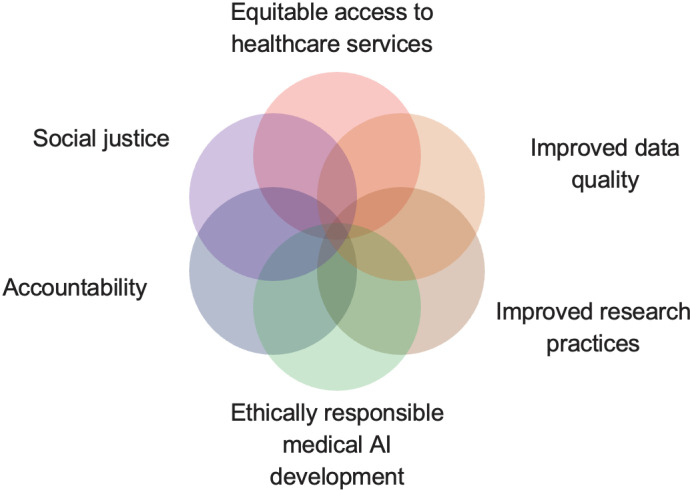
Perspectives from which this research was approached.

We however note, as a key limitation, that the authoring team’s research activity and clinical practice is predominantly U.S. based. Moreover, most members of the team are institutionally affiliated with a U.S. based research entity. To illustrate this point, the research clusters shown in Fig 1, represent the manner in which information flow, areas of focus and ideas are organized in the authoring team. They converge to L.C., who heads the Laboratory for Computational Physiology at the Massachusetts Institute of Technology and is central to the formation of the authoring team. This centrality of influence implies an inherent perspective skew towards U.S. institutional norms in the team’s perception of an effective TC. While the team has made efforts to incorporate diverse regional perspectives, this limitation, in our view, remains only partially mitigated.

### [5] Identity

The authoring team represents a diversity of cultural backgrounds and ages with appropriate gender representation. Self-reported ethnicities and cultural identities of our team members include: African-American, Arabian, East Asian, European, Igbo, Jewish American, KōKwel, Persian, Southeast Asian, Tswana, White American and White British. Members range in age from their 20s to their 50s, comprise self-identified men and women, and include members of the LGBTQIA+ community. Additionally, the team brings firsthand experience with the implementation of medical AI systems in clinical settings, as well as expertise across the development lifecycle of these systems, contributing to discussions on the need for greater accountability and diversity in AI research and development.

**Fig 2:**
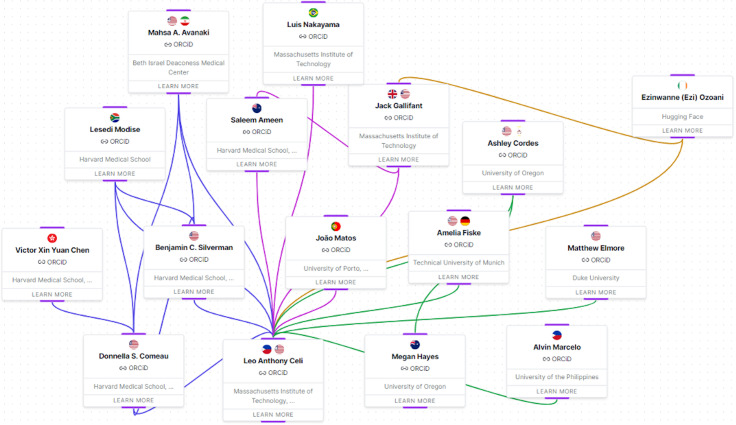
Relational network prepared by the authoring team. Our team’s relational network is visually represented as a series of spatial arrangements, communicating the overall team structure. Additionally, team members are interconnected with color-coded bands that denote functional clusters within the group. *Figure representing relationships among team members found at:* https://www.teamcard.io/team/demo. *It utilizes a forced-directed graph to display the multifaceted relationships and backgrounds of the team, in a clear, visually discernible way. The visualization was rendered using React Flow (a node-based graph visualization library), and the layout algorithm was implemented with the support of D3.js (a toolkit for data-driven documents). The graph consists of two fundamental components: nodes that represent team members, and edges that represent the relationships between team members. Prior to rendering, nodes were labeled according to their group affiliation, so that clusters of closely connected individuals can be displayed within close proximity to one another. After labeling, a structured circular layout for each cluster was implemented, such that each node within a cluster is evenly spaced to form a radial distribution of equal segments that are proportional to the number of nodes within the given cluster. This initial placement served as a starting point for the dynamic force simulation, which is implemented to iteratively refine node positions to an equilibrium state that represents the natural interconnections among team members.*

However, a key limitation is that the collective perspective of the team may be skewed toward that of a socioeconomic group with high levels of educational attainment and professional ties to prominent medical institutions. This bias is particularly relevant to the team’s perception of AI and its medical applications, which we acknowledge may be positively skewed. Consequently, the narrative presented in this manuscript could be enriched by the inclusion of socioeconomic perspectives that have less proximity, and differing levels of access, to the medical establishment. See Fig 2 for our team’s relational network.

We note that the inclusion of photographs with corresponding pronouns and LGBTQIA+ affiliation could improve the disclosure by providing a more complete visual representation of our social diversity. This level of disclosure has not been pursued by the team in recognition of the potentially sensitive nature of these attributes, due to the risk of potential discrimination. We have aggregated certain descriptors for the purposes of this illustration but could provide more granular disclosure to oversight bodies as needed. While efforts have been taken to balance dual commitments to individual privacy as well as to transparency and accountability, individual attributes that are not directly interpretable from the illustration prepared, could be vulnerable to reidentification in the context of subsequent TC disclosures.

## 7. Case studies

We further illustrate how the TC framework can be implemented for practical bias mitigation in the development of medical AI systems, through appropriately selected examples. We discuss cases of actual medical AI systems with identified bias, to demonstrate how the integration of the TC protocol could promote reflective development practices that better address and mitigate harmful biases. We also note that TCs are not intended to only be used in cases of demonstrated harm; all AI systems encode the possibility of harmful bias and can benefit from improved reflexivity among the teams developing these systems.

**Illustrative case 1: neurology - medical AI systems for the diagnosis of dementia**

Several systematic reviews of studies applying ML techniques to automatic speech and language processing for early dementia detection reveal that the use of speech datasets lacking in gender and age diversity is common practice in the field [81,82,83,84]. This reduces the accuracy and generalizability of resulting applications across demographic groups. Although there is recognition that commonly used dementia datasets should appropriately reflect the effects of age and gender on speech patterns [85], significant blind spots in the research persist. Key demographic factors that may impact dementia diagnosis—such as ethnicity, socioeconomic status, first language, and education level—are frequently overlooked in model training [84]. Additional challenges include an overrepresentation of Alzheimer’s disease relative to other dementia types in commonly used datasets [81], limited accent diversity in English-language datasets, insufficient language diversity, and a focus on languages primarily spoken in high-income regions [86]. Integrating TC protocols into research on ML techniques for automatic speech and language processing would support a shift away from standard practices with observed bias.

- Reflexivity: the reflective components of the TC protocol would be of particular relevance to the pre-processing and data compilation stages of the AI development lifecycle for dementia detection applications. Integrating the TC protocol would require research teams to openly reflect on the contextual factors shaping both the focus of their investigation and the datasets they select. This includes transparent discussions on data appropriateness and quality, identification of potential gaps, and actions taken to address these shortcomings.
- Representation: the protocol’s focus on appropriate representation in research teams would assist in surfacing often-overlooked factors, such as educational attainment and socioeconomic status, as well as culturally specific parameters like dialect, accent, and variations in syntax and intonation across languages. These variables significantly influence speech patterns and could impact the accuracy of automated dementia detection applications.
- End-user advocacy and ethical oversight: including patient advocate perspectives would enhance the interpretive resources available to researchers, allowing for the integration of important aspects of dementia’s lived experience into AI-powered early detection systems. These perspectives would help researchers better understand communication patterns in aging individuals, thereby mitigating the risk of unjust outcomes, such as misinterpretations of healthy speech patterns as signs of neurological decline.

**Illustrative case 2: facility management - algorithmic risk prediction in clinical settings**

Obermeyer et al [61]. demonstrate that algorithmic risk prediction tools commonly used in healthcare facilities across the U.S., result in substantial racial disparities in the program enrolment of over 200 million individuals annually. The tools are used to identify patients with complex care needs and accordingly, determine which should be enrolled in intensive care management programs. These tools have widely adopted historic health care spending, derived from insurance claim records, as a proxy for needed care. This inaccurately assumes that patients with lower historic healthcare costs are healthier than equally sick patients with higher spending and introduces a bias that favors White patients, who generally have better access to care and spend more on medical services than other racial groups in the U.S. This research underscores that algorithms designed to optimize healthcare expenditure can perpetuate racial biases, even when race is not explicitly observed as a variable. This occurs because the underlying problem definition — whether to prioritize the reduction of healthcare costs or to increase access to care — reflects and reinforces existing structural inequities embedded in historical practices [87].

Similarly, Sarkar et al. [65] find that clinical prediction models used during the COVID-19 pandemic to inform ICU triaging and decisions on the continuation of mechanical ventilation in resource-limited settings exhibit risk scoring biases across ethnic groups. Several commonly used models overpredict mortality in Black and Latino patients in the U.S., raising concerns about the disproportionate withholding or withdrawal of treatments (such as ventilators) in these demographics during the pandemic and in future crises.

These examples, along with other cases of algorithmic bias in clinical risk prediction, not only reflect but also exacerbate existing health inequalities by promoting unequal access to potentially life-saving treatments underscoring the need for a robust assurance protocol that could include the TC protocol and other prudential measures.

- Reflexivity: Since risk prediction algorithms are often applied to electronic health record (EHR) systems — where researchers may have limited control over data sources — the reflexive aspects of the TC protocol can help ensure greater scrutiny of algorithm design and specification. By incorporating the TC, research teams would need to assess the suitability of a given algorithm for EHR data, considering data quality, missing variables, and the potential impact of these limitations on predictive accuracy and bias. The protocol also encourages researchers to document their rationale for problem definition and the selection of proxy variables, reflect on how problem framing aligns with intended outcomes, evaluate how closely the proxies approximate the true variables of interest, and implement measures to mitigate any foreseeable risks. This approach promotes the more considered use of EHRs in algorithmic decision-making.
- Representation: A component of the harmful bias observed in risk prediction algorithms is attributable to researcher assumptions regarding what can be inferred from EHRs. Diverse representation within research teams can thus help bring to light potential limitations in these assumptions, particularly regarding how certain rules or variables may unintentionally exclude or disadvantage specific groups.
- Contextual testing and ethical review: Given the sensitive context in which clinical risk prediction algorithms are deployed, rigorous contextual testing prior to solution deployment is critical to the early identification of potential harmful bias. This testing should form part of an ethical review conducted by an IRB or a suitably qualified committee that can assess the social determinants influencing patterns in EHR data. An ethical review would assist researchers to identify and mitigate the effects of systemic inequality in available data, reducing the risk of deepening disparities in patient outcomes through algorithmic decision-making.

## 8. Considerations and limitations for operationalizing the TC and for ensuring diversity in the research and development of medical AI systems at scale

The TC’s practical approach to addressing unconscious bias in AI development through reflexivity has the potential to support a necessary cultural shift in the research and development of medical AI systems. While it has clear strengths, including its emphasis on ethical alignment, transparency, and accountability, its limitations – such as the lack of standard format and challenges to implementation – highlight areas for further refinement and adaptation.

### Key strengths of the protocol

- **Effective bias mitigation through reflexivity.** The primary strength of the TC protocol is that it serves to mitigate harm caused by unconscious bias, by putting the people behind the research and development of medical AI systems in transparent and reflective conversation with the performance of those systems in the field.
- **Practical integration of ethical considerations.** Another key strength of the protocol is that it integrates ethical considerations into AI research and development practices, serving to align the development of medical AI systems with established ethical standards for medical research [88].
- **Enhanced transparency and accountability**. By enhancing transparency regarding the potential biases embedded in medical AI systems, the TC protocol allows for more informed and discerning interaction with these products. This empowers physicians and other end users to act as more effective agents in mitigating discrimination by clinical algorithms. Thus, a further strength of the TC is that it aligns with the proposed regulation [9] for accountability in the development of medical AI systems.
- **Fair recognition of contributors.** By reporting on team composition with an emphasis on inclusion, the TC provides a practical mechanism for acknowledging the collective effort involved in the development of medical AI systems. It promotes the transparent recognition of contributors who might otherwise be overlooked and advocates for the inclusion of potential collaborators who might not traditionally be solicited for contribution.

### Key limitations of the protocol and possible mitigants

- **The lack of a standard format for TCs**. As the protocol is intended to be a transparent and informative expression of reflexivity in clinical AI research and the AI development process, the form and content of TCs may vary substantially across a diversity of applications and team structures in the ecosystem. While this flexibility has its advantages, it also challenges the establishment of fixed guidelines for TC reporting. As a mitigant, we note that positionality statements in qualitative research serve as a starting point in understanding the objectives and the necessary elements of a TC disclosure. From this basis, teams should interpret the cards as a conduit for self-reflection, acknowledgment of potential bias, and evaluation of the impact that any bias may have on the research under consideration. While the most appropriate format of this expression should be left to teams’ discretion, the content of the disclosure should respect a common understanding of the principles above. Additionally, asynchronous AI governance processes could prompt marked variation in the quality and the orientation of TC disclosures across regions. Here we believe that auxiliary initiatives to promote transparent, accurate, and useful reporting may include the implementation of validation protocols that are designed to identify and flag critical omissions by teams. One such validation protocol could be a medical AI development process that anticipates the formal review and approval of an IRB or an appropriately constituted ethics committee.
- **A required cultural shift.** TCs may be challenging to operationalize due to the voluntary and self-reported nature of the protocol. As the TC emphasizes epistemic practices—requiring self-reflection by the individuals behind medical AI systems regarding decisions about data inclusion, system performance, and development priorities—adoption may be inconsistent across the AI landscape, where reflective practices are often undervalued. Addressing this challenge necessitates a cultural shift toward reflexive approaches as essential components of bias mitigation. This shift can be facilitated by demonstrating the limitations of existing data and product focused protocols, such as Datasheets for Datasets, Model Cards and Algorithm Assurance, which currently offer only partial solutions to addressing bias in AI.
- **Privacy and integrity concerns.** The protocol may raise data privacy concerns and prompt pushback from team members regarding the public reporting of their personal information, with respect to unintended consequences such as potential tokenization and stigmatization. In response to this limitation, we maintain that while the TC is intended to promote greater transparency, it is nevertheless bound by standard privacy protection regulations such as the Health Insurance Portability and Accountability Act or the General Data Protection Regulation [89], which should allow teams to retain autonomy in their personal disclosures. Additionally, we contend that much of the information required for the TC is already disclosed by teams, through various professional and social media. However, this information is scattered across various platforms (e.g.,: websites, publications, presentations) and media (e.g.,: video and print) to little social benefit, whereas a protocol that centralizes all relevant disclosures by a given researcher, would greatly enhance transparency and accountability in the development of medical AI systems. The TC is thus an opportunity to consolidate existing metadata and to systematize the collection of further information. Future adaptations including integration of standard identifiers such as ORCID IDs in the protocol would serve to streamline the acquisition and evaluation of researcher information (ORCID is a non-profit organization that provides researchers with a unique digital identifier).
- **Practical impediments to implementation at scale.** We also note that the current scale of institutional research and development of medical AI systems presents a practical impediment to the implementation of the TC; certain disclosure parameters may become cumbersome to track where projects involve multiple teams collaborating across various stages of the AI development lifecycle. Here we might suggest multiple TCs for a single medical AI system, prepared in condensed and modular fashion, for discrete stages in the AI lifecycle. These disclosures could be accompanied by a clear record of the AI system’s progression, linking and narrating all relevant TC disclosures. The same approach could be adopted in instances where pre-existing models are leveraged by researchers, including instances in which open-source materials are used. The reflective elements of the TC module prepared for such cases would thus require a transparent discussion that interrogates the necessity and appropriateness of adopting pre-existing materials, selection criteria for these materials, as well as further considerations pertaining to possible implications for the research. These considerations should address the potential for legacy bias in the research and should consider actions to mitigate these effects.
- **The limitations of team diversity.** While we maintain that social diversity alone is insufficient to achieve the epistemic diversity necessary for effective bias mitigation in AI, it remains a valuable starting point for assembling orthogonal perspectives that can broaden the scope of inquiry and help identify potential blind spots. The TC provides a practical framework to facilitate this process by enabling teams to critically reflect on the positionality introduced by their composition and offers a system for tracking the relationship between diversity and epistemic outcomes, creating opportunities to better understand the value of diverse perspectives in AI research and development. We acknowledge that a focus on social diversity in teams has the potential to crowd out other priorities. This may be observed in instances where skill-based vetting of team members is revised to support diversity and inclusion. More broadly, existing social inequalities that perpetuate demographic skews in the market for talent in AI research and development will indeed limit the extent to which the social diversity promoted by the protocol can be operationalized in the landscape. Thus, in teams where there is a perceived lack of social diversity, the reflective elements of the protocol can focus on transparently acknowledging positionality skews within the team, considering their potential implications for the research – such as anticipated biases – and implementing measures to mitigate these risks. Tools such as Implicit Association Testing can further support this reflection by identifying unconscious biases that may otherwise go unnoticed but could influence the research outcomes.

## 9. Conclusion

The TC holds the promise of facilitating a deeper understanding of how diversity—both social and epistemic—shapes the development of ethical and robust medical AI systems. It provides concrete suggestions for embedding reflexivity into AI development workflows, equipping teams with tools to systematically document positionality variables and critically examine their influence on bias and decision-making.

This approach reframes the challenge of bias mitigation, shifting focus from narrow technical fixes to the subjective human dynamics that underlie AI design. Our experience with the TC underscores its potential to reveal and address the biases often encoded in medical AI systems, by fostering epistemic diversity and critical self-reflection in AI development practices.

As a prototype, the TC is a proof of concept—a tool designed to gather qualitative and quantitative data on team dynamics, with the goal of informing and refining future practices. Research from fields such as business, science, and organizational behavior provides compelling evidence that prioritizing diversity improves decision-making, fairness, and performance. The TC builds on this foundation, laying the groundwork for a cultural shift in AI research and development. Future iterations will expand its utility, adapting it to diverse contexts and scaling its impact to ensure inclusivity and accountability in the medical AI systems we build.
